# Subjective social status predicts long-term smoking abstinence

**DOI:** 10.1186/1471-2458-11-135

**Published:** 2011-02-25

**Authors:** Lorraine R Reitzel, Michael S Businelle, Darla E Kendzor, Yisheng Li, Yumei Cao, Yessenia Castro, Carlos A Mazas, Ludmila Cofta-Woerpel, Paul M Cinciripini, David W Wetter

**Affiliations:** 1 Department of Health Disparities Research, University of Texas MD Anderson Cancer Center, Houston, TX, USA; 2 Division of Health Promotion and Behavioral Sciences, University of Texas School of Public Health, Dallas Regional Campus, Dallas, TX, USA; 3 Department of Biostatistics, University of Texas MD Anderson Cancer Center, Houston, TX, USA; 4 Department of Behavioral Science, University of Texas MD Anderson Cancer Center, Houston, TX, USA

## Abstract

**Background:**

The relationship between subjective social status (SSS), a person's perception of his/her relative position in the social hierarchy, and the ability to achieve long-term smoking abstinence during a specific quit attempt is unknown. The purpose of this study was to examine the relationship between SSS and long-term smoking abstinence among 421 racially/ethnically diverse smokers undergoing a specific quit attempt, as well as the interactive effects of race/ethnicity and sex.

**Methods:**

The main effects and moderated relationships of SSS on biochemically-confirmed, continuous smoking abstinence through 26 weeks post-quit were examined using continuation ratio logit models adjusted for sociodemographics and smoking characteristics.

**Results:**

Even after adjusting for the influence of socioeconomic status and other covariates, smokers endorsing lower SSS were significantly less likely to maintain long-term smoking abstinence during a specific quit attempt than those with higher SSS (OR = 1.14, 95% CI: 1.00 - 1.28; *p *= 0.044). The statistical significance of this relationship, however, did not vary by race/ethnicity or sex.

**Conclusions:**

SSS independently predicts long-term smoking abstinence during a specific quit attempt. SSS may be a useful screener to identify smokers at elevated risk of relapse who may require additional attention to facilitate long-term abstinence. More research is needed to understand the mechanisms underlying the relationship between SSS and long-term smoking abstinence in order to appropriately tailor treatment to facilitate abstinence among lower SSS smokers.

## Background

Subjective Social Status (SSS) refers to the self-perception of one's status in the social hierarchy [1]. In general, those with greater financial resources typically endorse higher SSS. However, determinants of SSS extend beyond objective socioeconomic status (SES) indicators (such as income, education, and occupational status) to include satisfaction with financial resources, social trust, beliefs about upcoming opportunities, acculturation, and the anticipation of future security [2]. Although highly correlated with SES, several studies have indicated that SSS contributes unique variance in the prediction of self-rated health [3,4], health indicators [5], depression [4], negative affect [6], and health behaviors (e.g., fruit and vegetable consumption [7]). Most recently, research has shown that after adjusting for the effects of SES, higher SSS predicted greater rates of short-term smoking abstinence (i.e., through 2 weeks post-quit) during a smoking quit attempt [8]. Whether SSS predicts longer-term abstinence, however, is yet unknown. As smoking is becoming increasingly concentrated among individuals of low SES, incremental predictors of smoking cessation are useful to identify higher risk subgroups of smokers who may need more intensive interventions to successfully quit smoking.

SSS may represent an incremental predictor of various health-related outcomes over traditional indicators of SES because it captures the nuances of SES that might affect social standing (e.g., quality of education, prestige associated with a particular workplace, personal control in a particular job), taps into rarely assessed SES components (i.e., wealth), and captures the experience of societal inequalities and inequities [1,2,9,10]. These unique components of SSS may affect health-related outcomes through their associations with psychological distress (e.g., stress, depression) and physiologic dysfunction (in the case of low SSS) or psychological and physiological wellness (in the case of high SSS) [11]. They may also be particularly relevant to racial/ethnic minority groups or other segments of the population (e.g., women) that experience discrimination. Because these unique SSS components and their effects on the mechanisms underlying health-related outcomes may vary based on race/ethnicity and/or sex, the strength of the associations between SSS and health-related outcomes might also vary by race/ethnicity and/or sex. Indeed, racial/ethnic and gender differences have been cited in previous studies investigating associations between SSS and health outcomes [3,4,9]. However, although race/ethnicity and/or sex may moderate some SSS-health relationships, whether race/ethnicity and/or sex affect the relationship between SSS and smoking abstinence during a quit attempt is unknown. For a number of decades, smoking has remained the leading cause of preventable death and disability in the United States (U.S.) [12], and a clearer understanding of the factors predicting smoking cessation among historically understudied and underserved groups (e.g., racial/ethnic minorities, women) may facilitate the targeting of interventions to increase cessation rates, and help to identify groups in need of more intense interventions to quit smoking.

The purpose of this study was to examine the independent effect of SSS on long-term smoking abstinence during a specific quit attempt, while controlling for race/ethnicity, sex, SES, age, partner status, pre-quit smoking rate, and the number of years smoked. A complementary aim was to explore whether the relationship between SSS and long-term abstinence differed by race/ethnicity and/or sex. This study represents an extension of our earlier work linking SSS with short-term smoking abstinence (through 2 weeks post-quit) [8] to examine the effects of SSS on long-term smoking abstinence (through 26 weeks post-quit).

## Methods

### Procedures

Data for the current study were collected in Houston, Texas, as part of a longitudinal, cohort study designed to examine social disparities in smoking cessation [13],which was approved by the Institutional Review Board of The University of Texas MD Anderson Cancer Center. Houston is currently the 4^th ^largest city in the U.S. and the most populous of Texas' metropolitan statistical areas. The enrollment period for this study was from April 2005 - April 2007. Recruitment was via local print and radio advertisements. Inclusion criteria included: ≥21 years of age, daily smoker (≥5 cigarettes per day for the last year), motivated to quit in ≤30 days, and ≥6^th ^grade English proficiency. Potential participants were excluded if the nicotine patch was contraindicated, they reported use of tobacco products other than cigarettes, or they reported participation in a smoking cessation program within the past 90 days. Data for the current study were collected one week before quitting (baseline) and post-quit weeks 1, 2, 4, and 26.

### Participants

The parent study enrolled 424 racially/ethnically diverse participants. Sample size for the parent study was based on 80% power to detect a .25 standard deviation difference on questionnaire measures between any two racial/ethnic groups at any single point in time. All participants received standard smoking cessation treatment including six weeks of nicotine patch therapy, six brief smoking cessation counseling sessions based on the *Treating Tobacco Use and Dependence Clinical Practice Guideline*, [14] and self-help materials. Three participants in the parent study failed to answer the SSS item and were excluded from this study.

### Measures

All measures in this study were administered and completed via computer.

### Sociodemographic and Smoking Characteristics

Sociodemographic and smoking characteristics were measured at baseline and included race/ethnicity, sex, SES, age, partner status, pre-quit smoking rate (the number of cigarettes smoked per day), and the number of years smoked. Race/ethnicity was self-reported as: non-Latino White, non-Latino Black, or Latino. SES was represented by three variables: annual household income, educational level, and employment status. Categories of income were: <$20,000 a year or ≥$20,000. Categories of education were: < high school education, high school education (or GED), some college (no degree), or college degree. Categories of employment were: employed or not employed. Partner status categories were: married or living with partner versus other.

### Subjective Social Status

Subjective social status was measured at baseline with the SES version of the MacArthur Scale of Subjective Social Status, developed by the John D. and Catherine T. MacArthur Research Network on Socioeconomic Status and Health [15]. This measure presents a 10-rung ladder to the participant with instructions to imagine that it represents where people stand in society, with higher rungs representing higher status (i.e., more money, more education, and better jobs) [1]. Participants are asked to select the rung that best represents where they think they stand relative to others in the U.S., resulting in a continuous variable ranging from 1 to 10.

### Smoking Abstinence

Continuous abstinence from smoking was defined as a self-report of abstinence from smoking since the quit date (not even a puff), which was verified by expired carbon monoxide levels of ≤10 ppm. Smoking status was assessed at weeks 1, 2, 4, and 26 post-quit. Because the focus was on continuous abstinence, relapse at any post-quit week resulted in classification as relapsed from that point forward. The percentage of participants with missing smoking status was 18.7% at week 1, 16.1% at week 2, 14.3% at week 4, and 14.5% at week 26. An intention-to-treat procedure was followed, whereby participants with missing smoking status were considered not abstinent (i.e., relapsed).

### Data Analysis

All analyses were performed using Statistical Analysis Software (version 9.1). Preliminary analyses explored differences in sociodemographic and smoking characteristics by race/ethnicity and sex using Chi-Square tests for categorical variables and Analyses of Variance for continuous variables. Primary analyses examined the relationship between SSS and long-term abstinence. Because continuous abstinence was the outcome, continuation ratio (CR) logit models (SAS PROC GENMOD; [16-19]) were used to examine the influence of SSS on abstinence across weeks 1, 2, 4, and 26 post-quit. CR logit models are appropriate when ordered categories (e.g., relapsed at week 1, abstinent at week 1 but relapsed at week 2, abstinent at week 2 but relapsed at week 4, abstinent at week 4 but relapsed at week 26, and abstinent through week 26) represent a progression through stages [16-18]. The CR logit models operate by modeling the conditional probability of being abstinent at the current assessment point given that a participant has been abstinent through the most recent assessment point. Analyses adjusted for stage only were followed by analyses adjusted for stage and the following covariates: race/ethnicity, sex, SES, age, partner status, pre-quit smoking rate, and the number of years smoked. These covariates were selected based on their established relationships with smoking relapse (for example, see [20-22]), and included in order to isolate the effect of subjective social status on long-term, continuous abstinence over and above the effects of these variables. Next, additional adjusted CR models were used to examine whether race/ethnicity or sex (respectively) were potential moderators of the effect of SSS on long-term smoking abstinence.

## Results

### Sample Characteristics

Participants (*N *= 421, 46% male) were 33% non-Latino White (*n *= 137), 34% non-Latino Black (*n *= 144), and 33% Latino (*n *= 140). They were 41.2 years of age on average (*SD *= 11.2, median = 42, range 21-73), and 34% reported being married or living with a partner. With regard to SES variables, 38% of respondents reported less than $20,000 in annual household income, 14% lacked a high school diploma or equivalency, and 42% reported current unemployment. At baseline, participants smoked an average of 21.1 (*SD *= 10.3) cigarettes per day for an average of 21.6 years (*SD *= 11.1).

### Preliminary Analyses

Racial/ethnic groups were examined for significant differences on sociodemographics and smoking characteristics at baseline (Table 1). Results indicated that White participants smoked more cigarettes per day than both Black and Latino participants, and both Black and White participants smoked for more years than Latinos. Latinos were younger than the Black and White groups in this sample and more likely than the other racial/ethnic groups to be male, employed, and earning $20,000 or more in annual household income. Of all racial/ethnic groups, Black participants had the highest unemployment rates and the highest proportion of individuals earning less than $20,000 annually. There were no significant differences between racial/ethnic groups on SSS.

**Table 1:** Participants' Characteristics at Baseline by Race/Ethnicity

	White n = 137	Black n = 144	Latino n = 140	
	**n [%] or mean (±SD)**	**n [%] or mean (±SD)**	**n [%] or mean (±SD)**	***p *value**

**Demographics**				
Sex				0.022
Female	78 [56.9]	86 [59.7]	62 [44.3]	
Male	59 [43.1]	58 [40.3]	78 [55.7]	
Age	42.9 (±11.7)	44.8 (±9.9)	36 (±10.2)	<0.001
Partner Status				<0.001
Married/living with partner	42 [30.9]	35 [24.5]	67 [48.6]	
Other (e.g., single)	94 [69.1]	108 [75.5]	71 [51.4]	

**Socioeconomic Status**				
Annual Household Income				<0.001
<20,000	47 [39.2]	76 [57.1]	35 [29.4]	
≥20,000	73 [60.8]	57 [42.9]	84 [70.6]	
Educational Level				0.009
<High school	14 [10.3]	17 [11.9]	27 [19.6]	
High school/GED	29 [21.3]	29 [34.3]	42 [30.4]	
Some college (no degree)	43 [31.6]	44 [30.8]	41 [29.7]	
College degree	50 [36.8]	33 [23.0]	28 [20.3]	
Employment Status				<0.001
Employed	71 [52.6]	68 [47.9]	101 [73.0]	
Unemployed	64 [47.4]	74 [52.1]	37 [26.8]	

**Smoking Characteristics**				
Average number of CPD	23.4 (±9.1)	20.9 (±12.0)	19 (±9.0)	0.001
Number of years smoked	23.2 (±11.8)	23.5 (±11.3)	18 (±9.3)	<0.001

**Social Status Ladder**	6.0 (±1.9)	5.8 (±1.7)	6.0 (±1.8)	0.507

Note: CPD = Self-reported number of cigarettes smoked per day at baseline. GED = General Equivalency Diploma.

Sex groups were also examined for significant differences on sociodemographics and smoking characteristics at baseline (Table 2). Results indicated that women smoked fewer cigarettes per day than men prior to quitting. Notably, women were significantly more likely to endorse an annual household income of less than $20,000 and to be unemployed. Women also endorsed lower SSS than men.

**Table 2:** Participants' Characteristics at Baseline by Sex

	Men n = 195	Women n = 226	
	**n[%] or mean (±SD)**	**n[%] or mean (±SD)**	***p *value**

**Demographics**			
Race/ethnicity			0.022
Non-Latino White	59 [30.0]	78 [34.5]	
Non-Latino Black	58 [29.7]	86 [38.1]	
Latino	78 [40.0]	62 [27.4]	
Age	41.2 (±11.3)	42.3 (±11.2)	0.888
Partner Status			0.212
Married/living with partner	72 [37.7]	72 [31.9]	
Other (e.g., single)	119 [62.3]	154 [68.1]	

**Socioeconomic Status**			
Annual Household Income			0.061
<20,000	65 [37.4]	93 [47.0]	
≥20,000	109 [62.6]	105 [53.0]	
Educational Level			0.433
<High school	27 [14.1]	31 [13.7]	
High school/GED	62 [32.5]	58 [25.6]	
Some college (no degree)	56 [29.3]	72 [31.9]	
College degree	46 [24.1]	65 [28.8]	
Employment Status			0.005
Employed	124 [65.3]	116 [51.6]	
Unemployed	66 [34.7]	109 [48.4]	

**Smoking Characteristics**			
Average number of CPD	23.1 (±11.8)	19.4 (±8.4)	<0.001
Number of years smoked	21.9 (±11.1)	21.3 (±11.2)	0.627

**Social Status Ladder**	6.1 (±1.7)	5.7 (±1.8)	0.018

Note: CPD = Self-reported number of cigarettes smoked per day at baseline. GED = General Equivalency Degree.

### Primary Analyses

In unadjusted analyses, SSS predicted long-term smoking abstinence [*β *= .20, SE = .05; χ^2 ^(1) = 14.83; OR = 1.23 (95% CI: 1.11-1.36); *p *= .0001]. In analyses controlling for race/ethnicity, sex, SES, age, partner status, pre-quit smoking rate, and the number of years smoked, SSS remained a significant predictor of long-term smoking abstinence [*β *= .13, SE = .06; χ^2 ^(1) = 4.07; OR = 1.14, 95% CI: 1.00 - 1.28; *p *= .044; Table 3]. The SSS by stage interaction was not significant, indicating that the effect of SSS on abstinence was consistent across post-quit weeks 1, 2, 4, and 26 (*p *= .145).

**Table 3:** Results of Unadjusted and Adjusted Models for Subjective Social Status Predicting Smoking Abstinence

Variables in Unadjusted Model	OR	95% CI
**Social Status Ladder**	**1.23**	**1.11-1.36**

**Variables in Adjusted Model**	**OR**	**95% CI**

Race/ethnicity		
Non-Latino White	1	
Non-Latino Black	0.90	0.54-1.53
Latino	0.98	0.58-1.67
Sex		
Male	1	
Female	1.09	0.73-1.64
Age	1.04	1.01-1.07
Partner Status		
Married or living with partner	1	
Other (e.g., single)	0.82	.053-1.28
Annual Household Income		
<20,000	0.79	0.48-1.31
≥20,000	1	
Education Level		
<High school	1	
High school/GED	1.00	0.51-1.97
Some college (no degree)	1.20	0.63-2.30
College degree	0.97	0.49-1.91
Employment Status		
Employed	1	
Unemployed	0.51	0.31-0.85
Average number of CPD	1.01	0.98-1.03
Number of years smoked	0.99	0.97-1.02
**Social Status Ladder**	**1.14**	**1.00-1.28**

Note: OR = Odds Ratio. CPD = Self-reported number of cigarettes smoked per day at baseline. GED = General Equivalency Degree. Both unadjusted and adjusted models controlled for stage.

### Moderator Analyses

The relationship between SSS and long-term abstinence was not moderated by race/ethnicity or sex in unadjusted [race/ethnicity: χ^2 ^(2) = 1.94, *p *= .379; sex: χ^2 ^(1) = 0.28, *p *= .597] or adjusted [race/ethnicity: χ^2 ^(2) = 0.56, *p *= .755; sex: χ^2 ^(2) = 0.01, *p *= .924] analyses.

## Discussion

The purpose of this study was to examine whether SSS predicted long-term smoking abstinence (through 26 weeks post-quit) among a racially/ethnically diverse sample of smokers undergoing a specific quit attempt. The results demonstrated that SSS conveys unique predictive information with respect to long-term smoking abstinence, with higher SSS being associated with a greater likelihood of maintaining abstinence over time. While there have been a number of cross-sectional studies linking SSS with self-rated health and health indicators, few studies have used a longitudinal design and even fewer have done so in the examination of the effect of SSS on health behaviors. The current research builds on our previous work demonstrating that SSS predicted short-term smoking abstinence in this racially/ethnically diverse sample [8], and suggests that quitting smokers endorsing lower SSS may face significant hurdles in maintaining both short- and long-term abstinence. These results add to a growing body of literature documenting that SSS contributes to the prediction of self-rated health [3,4], health indicators [4-6], and health behaviors [7] over and above the influence of objective SES indicators.

A secondary aim of this study was to assess whether race/ethnicity and/or sex moderated the relationship between SSS and long-term smoking abstinence. Although previous studies have supported that relationships between SSS and health-related outcomes are moderated by race/ethnicity and sex [3,4,9], this study failed to support the moderating effects of these variables on the relationship between SSS and long-term smoking abstinence. One possible explanation for this finding is that the unique factors that SSS captures over SES are common to all population groups examined, and therefore affected smoking abstinence equally among these groups. However, this may be unlikely given prior research indicating that determinants of SSS differ by race/ethnicity and sex [3,4,9] and, in some cases, may be relevant only to certain racial/ethnic groups (e.g., acculturation [23]). Therefore, another explanation might be that the unique components of SSS, although varying in significance and weighted differently between race/ethnicities and the sexes, have a similar effect on the mechanisms underlying smoking cessation. Many studies have confirmed the role of depression, negative affect, and stress in smoking relapse (e.g., [24-27]), and it may be that these are the mechanisms through which SSS uniquely predicts smoking abstinence among all racial/ethnic and sex groups. This supposition is partially supported by previous research finding that depression mediated the relationship between SSS and short-term smoking abstinence [8]. However, a post-hoc analysis indicated that further adjusting our models for baseline depressive symptoms as measured by the Center for Epidemiologic Studies Depression Scale [28] did not alter the pattern of results. A better understanding of the mechanisms underlying the relationship between SSS and smoking cessation, and how these may vary over the course of the quit attempt, is needed.

Strengths of this study include the longitudinal design and the use of a racially/ethnically diverse sample of participants. Because smoking is the leading cause of morbidity and mortality in the U.S. and is a major contributor to health disparities [12], enhancing understanding of the predictors of smoking abstinence is vital to the development of interventions and the targeting of treatment to affected groups. Results of the current study clearly demonstrate that smokers endorsing lower SSS are at increased risk of relapse during a smoking quit attempt as compared with smokers endorsing higher SSS scores. Results also suggest that individuals endorsing lower SSS might benefit from targeted interventions to help facilitate smoking cessation. Whether such targeted interventions might include a more intense (e.g., greater coverage of issues) or higher dosage (e.g., increased number of sessions) intervention for smoking cessation than standard treatment is fodder for future research. However, the standard smoking cessation intervention provided to all participants in this study (i.e., brief counseling, self-help materials, and the "patch") was not sufficient to enable cessation among lower SSS smokers in this study. As the SSS scale is a relatively quick and easy tool to administer [15], smoking cessation programs could use it as a screener for smokers who may require additional attention to facilitate abstinence, although more information is needed on what might constitute a meaningful SSS cut-off for additional service provision. Future research should explore how to better treat lower SSS smokers, which would benefit from a greater understanding of the mechanisms driving the relationship between SSS and smoking abstinence.

Another potential application of this finding could entail the development of interventions designed to facilitate smoking cessation while also affecting the perceived social status of lower SSS individuals. This is a completely unexplored area but might entail building social trust [2,10] by encouraging involvement in community or church activities, enhancing social standing through the adoption of community leadership roles (e.g., homeowners association board, neighborhood watch), or facilitating hope for the future by assisting in the identification and utilization of local resources (e.g., job placement agencies, food banks, reduced-rate health care options). It is unknown whether such interventions might affect SSS and facilitate smoking cessation, but these possibilities could be examined in future research.

Limitations of the current study include the potential for limited power to detect interaction effects, as the parent study was not specifically designed to explore moderators of SSS effects on long-term cessation. Future studies should replicate these analyses with a more purposeful design. Future studies in this area might also adjust for other SES variables not collected (e.g., wealth) in the present study in order to further isolate the unique effects of SSS on health indicators and outcomes. Also, participants in this study were self-selected, treatment-seeking smokers who may differ from smokers who attempt to quit without treatment in important ways, and the influence of SSS on cessation among the latter group remains unknown. It is also worthy of note that this study relied on self-report of racial/ethnic group membership, which may not perfectly align with biologically-based classifications of race/ethnicity. Also, the parent study was designed to recruit Whites, Blacks and Latino smokers in equal proportions, which may not be representative of the local population in terms of racial/ethnic distribution or other factors. Thus, results may not generalize to the larger population of smokers in Houston. Importantly, our interpretation of the data assumes that the unique predictive ability of SSS over SES indicators is not based on measurement error. Although these assumptions have been supported in previous literature (e.g., [1,29]), we cannot rule out that the presence of unknown and unmeasured confounders might have influenced these results.

## Conclusions

This study examined whether SSS predicted long-term abstinence from smoking during a specific quit attempt, and assessed potential moderation by racial/ethnic group or sex. Our results indicated that smokers endorsing higher SSS were more likely to maintain long-term smoking abstinence during a specific quit attempt than smokers with lower social status ratings, regardless of their race/ethnicity or sex. Although a number of studies have supported inverse relationships between SES indicators (e.g., income, education) and smoking cessation (cf. [13]), the current study indicates that SSS is an even stronger predictor. SSS, the self-reported perception of relative standing in the social hierarchy, captures SES but also taps into the experience of societal inequities and a person's feelings of hope about future status change [1,10]. It may be these, or other, unique components of SSS that are especially important influences on health and health behaviors. More research is needed to understand the mechanisms underlying the relationship between SSS and long-term smoking abstinence, and to appropriately tailor treatment to facilitate abstinence among smokers endorsing lower SSS.

## Competing interests

The authors do not have any competing interests directly pertaining to this work; however, we would like to report that PM Cinciripini has served on the scientific advisory board of Pfizer Pharmaceuticals and has conducted educational talks for physicians on smoking cessation that were sponsored by Pfizer within the past five years.

## Authors' contributions

LRR, MSB, DEK, YCastro, and DWW conceptualized the research question and wrote the manuscript. YL and YCao conducted the data analysis, interpreted results, and reviewed the data analysis and results sections. DWW is the senior author of the paper, and the principal investigator on the supporting grant (National Institute of Drug Abuse; R01DA014818). CAM, LC-W, and PMC helped with the conceptualization of the overall project and methodology, and reviewed and edited manuscript drafts. PMC is a co-investigator on the supporting grant. All authors have read, contributed to, and approved the final manuscript.

## Pre-publication history

The pre-publication history for this paper can be accessed here:

http://www.biomedcentral.com/1471-2458/11/135/prepub
